# Cell-free microRNA expression signatures in urine serve as novel noninvasive biomarkers for diagnosis and recurrence prediction of bladder cancer

**DOI:** 10.18632/oncotarget.16586

**Published:** 2017-03-28

**Authors:** Lutao Du, Xiumei Jiang, Weili Duan, Rui Wang, lishui Wang, Guixi Zheng, Keqiang Yan, Lili Wang, Juan Li, Xin Zhang, Hongwei Pan, Yongmei Yang, Chuanxin Wang

**Affiliations:** ^1^ Department of Clinical Laboratory, Qilu Hospital of Shandong University, Jinan, 250012, Shandong Province, China; ^2^ Department of Urology, Qilu Hospital of Shandong University, Jinan, 250012, Shandong Province, China; ^3^ Department of Clinical Laboratory, The Second Hospital of Shandong University, Jinan 250033, Shandong, China

**Keywords:** microRNA, bladder cancer, diagnosis, recurrence, urine

## Abstract

Urinary microRNAs (miRNAs) are potential biomarkers for the noninvasive diagnosis of bladder cancer (BC). In this study, we aimed to develop a urinary miRNAs panel for diagnosing and predicting recurrence of BC. Genome-wide miRNAs analysis by deep sequencing followed by two phases of quantitative real-time PCR assays were performed on urine supernatant of 276 BC patients and 276 controls. We identified a seven-miRNA panel (miR-7-5p, miR-22-3p, miR-29a-3p, miR-126-5p, miR-200a-3p, miR-375, and miR-423-5p) that provided high diagnostic accuracy of BC with an AUC of 0.923 and 0.916 in training and validation set, respectively. The corresponding AUCs of this panel for Ta, T1 and T2-T4 were 0.864, 0.930 and 0.978, significantly higher than those of urine cytology, which were 0.531, 0.628 and 0.724, respectively (all p < 0.05). Moreover, Kaplan–Meier analysis showed that nonmuscle-invasive BC (NMIBC) patients with high miR-22-3p and low miR-200a-3p level had worse recurrence-free survival (RFS) (p = 0.002 and 0.040, respectively). Multivariate Cox regression analysis revealed that miR-22-3p and miR-200a-3p were independently associated with RFS of NMIBC (p = 0.024 and 0.008, respectively). In conclusion, our results suggested that urinary miRNAs may have considerable clinical value in diagnosis and recurrence prediction of BC.

## INTRODUCTION

Bladder cancer (BC) is one of the most common and lethal urological malignances worldwide. The incidence of BC has substantially increased over the last 10 years, and 74690 new cases were diagnosed [1]. The major problem of BC is the high recurrence rate and the recurrence of more than half of these cases can be observed within 5 years [2]. Therefore, early screening and monitoring should be the essential to improving treatment outcomes for patients with BC. Currently, cystoscopy is considered as the gold standard for the diagnosis of BC, but it is invasive and expensive. Voided urine cytology is non-invasive, but it has low sensitivity to diagnose low-grade BC. Many urine-based biomarkers such as nuclear matrix protein 22 (NMP22), bladder tumor antigen (BTA) and cytokeratin are still in the progression of continuous development during the past decades, but no one is ideal and cannot be recommended for large-scale cancer screening [3]. Therefore, novel biomarkers for diagnosing BC, especially at the early stage, and monitoring the recurrence of BC with high sensitivity and specificity should be explored.

MicroRNA (miRNA) is a class of small non-coding RNA of 19-25 nucleotides in length. They negatively regulate gene expression at the post-transcriptional level and participate in almost all of the known hallmarks of oncogenesis and tumor metastasis [4–7]. Previous studies have shown the existence of a large amount of stable miRNAs in human urine, and laid the foundation for studying the role of urinary miRNAs in diagnosis of BC [8–10]. Differential expression of miRNAs in urine has been reported recently [11–14]. However, these studies had small sample size and limited number of screened miRNA. In addition, they either were performed without suitable reference gene, or were independent validation, and therefore failed to identify unique miRNA profiles.

In the present study, we performed high-throughput next generation sequencing using the Illumina MiSeq platform, followed by confirmation with two phases of RT-qPCR assays to systematically and extensively characterize the cell-free miRNA-production profile in urine of patients with BC. We first identified the most suitable reference genes for urine miRNA detection in BC. We then determined that a seven-miRNA panel might serve as a novel diagnostic indicator for BC. Moreover, correlation between the seven miRNAs and tumor recurrence was further assessed.

## RESULTS

### Urinary miRNA expression profiles of BC by MiSeq sequencing

By MiSeq sequencing, a total of 498 miRNAs with at least 20 copies were scanned. Among these miRNAs, 256 and 308 miRNAs with at least 50 copies were detected in controls and BC patients, respectively. To identify potential reference miRNAs for BC, we selected 13 miRNAs showed lower than 1.2-fold changes with no significant differences between the two groups (each *p* > 0.05). The miRNAs were considered as altered only if the absolute fold change was significantly larger than 2-fold between BC and control groups. Using the above criterion, 13 miRNAs (each *p* > 0.05, Supplementary Tables 1) were determined as candidate reference gene and 23 miRNAs were differentially expressed between the two groups (each *p* < 0.05, Supplementary Table 2).

**Table 1:** The relative expression of selected urinary miRNA in patients with BC and controls in training set and validation set [median (interquartile range)]

miRNA	Training set			Validation set		
	Controls (n=150)	BCs (n=150)	*p* Value	Controls (n=120)	BCs (n=120)	*p* Value
miR-22-3p	1.14 (0.54-1.88)	2.91 (1.83-4.05)	<0.001	1.01 (0.53-1.97)	2.76 (1.65-3.98)	<0.001
miR-29a-3p	1.34 (0.54-2.71)	2.60 (1.18-6.53)	<0.001	1.14 (0.50-2.11)	1.76 (0.77-4.17)	<0.001
miR-375	1.10 (0.73-1.71)	2.07 (1.24-3.73)	<0.001	1.03 (0.70-1.51)	1.83 (1.18-2.61)	<0.001
miR-7-5p	0.92 (0.49-2.21)	1.43 (0.86-2.73)	<0.001	1.00 (0.56-1.80)	1.44 (0.80-2.81)	<0.001
miR-126-5p	1.29 (0.47-1.86)	3.12 (1.01-5.20)	<0.001	1.12 (0.68-1.62)	2.37 (1.00-4.26)	<0.001
miR-423-5p	1.04 (0.52-1.94)	0.47 (0.20-0.93)	<0.001	1.01 (0.53-1.75)	0.54 (0.23-1.20)	<0.001
miR-200a-3p	1.07 (0.54-1.82)	0.62 (0.34-0.91)	<0.001	0.94 (0.54-1.87)	0.62 (0.31-1.05)	<0.001

### Identification of suitable reference genes for BC

To identifysuitable endogenous controls for normalizing cell-free miRNAs specific to urine in BC, the 13 candidate reference miRNAs and U6 were subjected to RT-qPCR assays using a cohort of 80 BC patients and 80 controls, because U6 was commonly used for expression normalization. The Cq values were determined using the default threshold setting. The miRNAs with a Cq value > 35 and detection rate < 75% in either BC group or control group were excluded from further analyses. Eight reference genes (let-7b-5p, miR-20a-5p, miR-23b-5p, miR-28-3p, miR-34a-5p, miR-100-5p, miR-532-5p and U6) passed the quality control process and there was no evidence for differential expression of these genes between BCs and controls (all *p* > 0.05, Supplementary Figure 1). Variable stability of selected reference miRNAs were evaluated using geNorm and NormFinder. The two algorithms both identified let-7b-5p as the most stably expressed reference gene, and selected let-7b-5p and miR-532-5p as the most stable pair of reference genes(Supplementary Table 3, Supplementary Figure 2). To further validate the stability of the identified reference genes, we applied another cohort (63 BC patients and 63 controls). Based on the Cq value of each validate reference gene, there was no evidence of differential miRNA expression between BCs and controls at different stages of BC (Supplementary Figure 3).

### RT-qPCR analysis of differentially expressed miRNAs in BC

**Figure 1:**
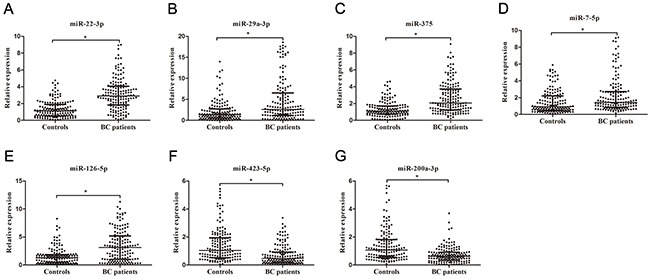
Relative expression of seven selected miRNAs in urine Relative expressions of seven selected urinary miRNAs in patients with BC (n = 150) and control individuals (n = 150) using RT-qPCR assay in training set **(A–G)**, *p< 0.001.

Using miR-532-5p and let-7b-5p as the reference genes, we next used RT-qPCR assay to confirm the expressions of 23 candidate miRNAs which were selected from the previous step. Only miRNAs with statistically significant expression (*p* < 0.001) were selected from the training set for further validation. RT-qPCR analysis revealed that five miRNAs (miR-7-5p, miR-22-3p, miR-29a-3p,miR-126-5p, and miR-375) were up-regulated and two (miR-200a-3p, miR-423-5p) were down-regulated in BCs (Table 1, Figure 1). The diagnostic performance of the seven miRNAs was evaluated by ROC analysis. The AUCs of these miRNAs were 0.639, 0.803, 0.67, 0.705, 0.748, 0.692, and 0.72, respectively (Supplementary Figure 4). In the validation cohort, the expressions of these seven miRNAs were further measured by RT-qPCR assay. The expressions of the seven miRNAs in the validation set were consistent with those in the training set (Table 1, Supplementary Figure 5).

### Establishment and validation of the predictive diagnostic miRNAs panel

**Figure 2:**
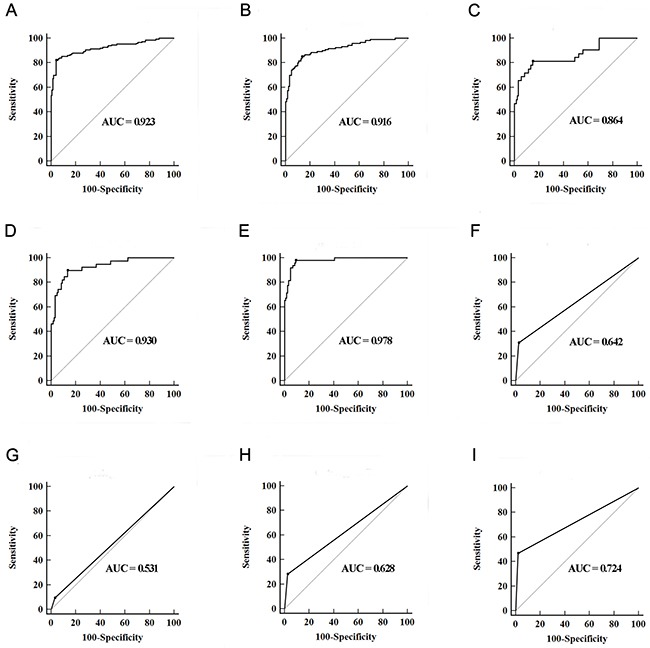
Diagnostic performance of three-lncRNA panel and urine cytology for the detection of BC **(A and B)** ROC curves for the detection of BC using seven-miRNA panel in training set (A) and validation set (B); **(C–E)** ROC curves using the seven-miRNA panel for the detection of Ta (C), T1 (D) and T2-T4 (E) in validation set; **(F–I)** ROC curves analysis using urine cytology for the detection of BC with all stages (F), Ta (G), T1 (H) and T2-T4 (I) in validation set.

In the training set, a stepwise logistic regression model was constructed for diagnosis of BC as follows: logit (*p* = BC) = -0.7792 – (0.0823×miR-7-5p) – (0.2015×miR-22-3p) – (0.0223×miR-29a-3p) – (0.0793×miR-126-5p) + (0.1522×miR-200a-3p) – (0.1545×miR-375) + (0.2234×miR-423-5p). ROC analysis revealed that the AUC of the miRNA panel was 0.923 (95% confidence interval [CI], 0.886 to 0.950, Figure 2A). The sensitivity of the miRNA panel was 82.00% and the specificity was 96.00%.

In the validation set, the ability to predict BC by the constructed seven-miRNA panel was further assessed. The AUC of the miRNA panel was 0.916 (95% CI, 0.873 to 0.948, sensitivity =85.00%, specificity = 86.67%, Figure 2B). The AUCs of the panel for Ta, T1 and T2-T4 were 0.864 (sensitivity = 81.25%, specificity = 85.00%), 0.930 (sensitivity = 89.74%, specificity = 86.67%) and 0.978 (sensitivity = 97.78%, specificity = 90.83%), respectively (Figure 2C-2E). The AUCs of the panel for low grade BC and high grade BC were 0.911 (sensitivity = 80.88%, specificity = 91.67%) and 0.958 (sensitivity = 94.23%, specificity = 89.17%), respectively. Meanwhile, urine cytology was performed to compare the diagnostic performance of the seven-miRNA panel. The AUC of urine cytology was 0.642 (95% CI, 0.577to 0.702, sensitivity = 30.83%, specificity = 97.50%, Figure 2F). The corresponding AUCs of this panel for Ta, T1 and T2-T4 were significantly higher than those of urine cytology, which were 0.531, 0.628 and 0.724, respectively (all at *p* < 0.05, Figure 2G-2I).

### Correlation between the expression levels of the seven miRNAs and clinicopathological characteristics

As shown in Table 2, high level of miR-22-3p and miR-375 expression, along with low level of miR-423-5p expression significantly correlated with advanced tumor stage (*p* = 0.02, *p* = 0.03, and *p* = 0.03, respectively). High level of miR-29a-3p and miR-375 expressions correlated with positive lymph node metastasis, and a higher level of miR-7-5p expression correlated with a higher tumor grade (*p* = 0.03, *p* = 0.04, and *p* = 0.02, respectively). However, there were no significant associations between the expression levels of the seven miRNAs and age or sex (all at *p* > 0.05).

**Table 2:** Correlations between urinary miRNA concentrations and clinicopathological characteristics of patients with BC in validation set [median (interquartile range)]

Parameters	Total cases	miR-22-3p	*p*	miR-29a-3p	*p*	miR-375	*p*	miR-7-5p	*p*	miR-126-5p	*p*	miR-423-5p	*p*	miR-200a-3p	*p*
**Age**			0.26		0.92		0.22		0.45		0.34		0.10		0.11
<65	57	2.70 (1.80-3.82)		1.83 (1.08-4.28)		1.88 (1.38-2.69)		1.52 (0.95-2.79)		3.01 (1.40-4.05)		0.52 (0.29-0.90)		0.73 (0.35-1.06)	
≥65	63	3.06 (1.94-4.70)		2.20 (0.76-5.00)		1.69 (1.04-2.47)		1.26 (0.67-3.10)		2.03 (0.88-4.70)		0.35 (0.15-0.89)		0.50 (0.26-1.01)	
**Sex**			0.20		0.37		0.82		0.10		0.08		0.14		0.17
Male	94	3.06 (1.84-4.29)		1.92 (0.96-4.11)		1.77 (1.17-2.63)		1.37 (0.69-2.75)		2.10 (0.90-3.90)		0.42 (0.17-0.94)		0.54 (0.29-0.94)	
Female	26	2.58 (1.75-3.63)		3.48 (0.99-5.61)		1.90 (1.28-2.54)		1.71 (1.07-2.98)		3.36 (1.50-4.86)		0.53 (0.37-0.84)		0.72 (0.46-1.14)	
**Tumor stage**			0.02		0.54		0.03		0.44		0.09		0.03		0.73
Ta-T1	71	2.59 (1.46-4.01)		2.02 (0.76-4.18)		1.52 (1.06-2.56)		1.27 (0.77-3.10)		1.74 (0.88-4.23)		0.52 (0.23-1.15)		0.59 (0.29-1.05)	
T2-T4	49	3.21 (2.20-4.74)		2.35 (1.10-4.58)		2.16 (1.39-3.03)		1.52 (1.00-2.79)		3.20 (1.40-4.31)		0.40 (0.14-0.71)		0.65 (0.35-1.05)	
**Tumor grade**			0.16		0.52		0.10		0.02		0.07		0.24		0.17
Low grade	46	3.18 (2.00-4.68)		1.98 (0.73-5.11)		1.61 (0.56-2.59)		1.09 (0.65-2.21)		1.53 (0.82-3.92)		0.41 (0.16-0.82)		0.54 (0.26-0.94)	
High grade	74	2.71 (1.61-3.84)		2.16 (1.14-4.27)		1.88 (1.33-2.67)		1.61 (1.01-3.09)		3.02 (1.45-4.29)		0.47 (0.24-0.93)		0.69 (0.36-1.06)	
**Lymph node metastasis**			0.80		0.03		0.04		0.96		0.83		0.84		0.61
Negative	104	3.01 (1.83-4.06)		1.81 (0.83-3.99)		1.68 (1.14-2.56)		1.47 (0.79-2.81)		2.37 (1.00-4.30)		0.44 (0.18-0.92)		0.60 (0.34-1.05)	
Positive	16	2.47 (1.69-4.33)		4.21 (1.20-9.88)		2.44 (1.30-4.19)		1.39 (0.90-2.87)		2.58 (0.84-4.19)		0.40 (0.23-0.81)		0.65 (0.26-1.04)	

### Restoration of the expressions of the seven miRNAs in postoperative patients

To demonstrate the crucial link between BC status and the deregulated miRNAs, the expressions of the seven miRNAs were quantified in the preoperative and postoperative urine samples from 21 BC patients in the validation phase. Urinary miR-22-3p and miR-29a-3p expressions were significantly down-regulated after surgery in the same subset of patients (*p* = 0.015 and *p* = 0.017, respectively, Supplementary Figure 6). However, there were no significant differences in the expression levels of other miRNAs before and after surgery (all at *p* > 0.05).

### Identification of potential prognostic factors for the recurrence of BC

**Figure 3:**
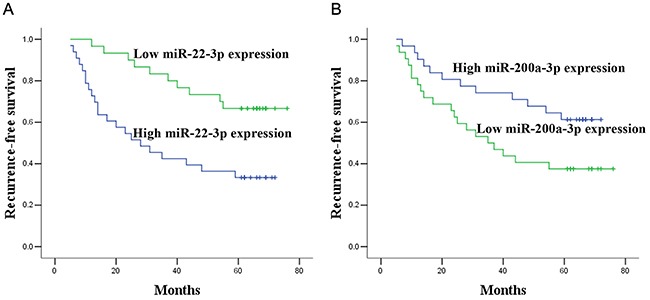
Prognostic significance of miR-22-3p and miR-200a-3p expression Kaplan–Meier curves for recurrence-free survival according to the urine levels of miR-22-3p (A) and miR-200a-3p (B) in NMIBC patients in validation set.

In the validation cohort, survival analysis was performed in NMIBC group and MIBC group, respectively. In the NMIBC group, according to the Kaplan–Meier curve, patients with high miR-22-3p levels and low miR-200a-3p levels had dramatically lower recurrence-free survival (RFS) than those with low miR-22-3p levels and high miR-200a-3p levels (*p* = 0.002, and *p* = 0.040, respectively, Figure 3). Univariate Cox proportional hazards regression model analysis revealed a significant correlation between recurrence and miR-22-3p (*p* = 0.004), miR-200a-3p (*p* =0.045) and tumor stage (*p* = 0.006). Parameters significantly related to RFS in the univariate analysis were then put into the multivariate analysis to identify independent factors for prognoses of NMIBC. Multivariate analysis showed that miR-22-3p, miR-200a-3p and tumor stage maintained their significance for recurrence of NMIBC (*p* = 0.024, *p* = 0.008, and *p* = 0.008, respectively, Table 3). In the MIBC group, there were no miRNAs that influenced patient predicted recurrence (all at *p* > 0.05, Supplementary Table 4).

**Table 3:** Univariate and multivariate Cox proportional hazards regression model analysis of recurrence-free survival in NMIBC patients in validation set

Parameters	Categories	Univariate analysis		Multivariate analysis		
		HR (95% CI)	*p* Value	Parameters	HR (95% CI)	*p* Value
age	<65 vs. ≥65	0.816 (0.408-1.632)	0.565			
sex	Male vs. Female	1.220 (0.548-2.717)	0.626			
Tumor stage	Ta vs. T1	2.852 (1.346-6.043)	0.006	Tumor stage	2.956 (1.324-6.602)	0.008
Tumor grade	Low vs. High	0.712 (0.450-1.126)	0.147			
Lymph node metastasis	Negative vs. Positive	0.541 (0.074-3.966)	0.546			
miR-22-3p expression	Low vs. High	3.040 (1.435-6.443)	0.004	miR-22-3p expression	2.469 (1.128-5.406)	0.024
miR-29a-3p expression	Low vs. High	1.206 (0.599-2.425)	0.600			
miR-375 expression	Low vs. High	0.969 (0.484-1.943)	0.930			
miR-7-5p expression	Low vs. High	0.677 (0.337-1.363)	0.275			
miR-126-5p expression	Low vs. High	0.725 (0.362-1.452)	0.365			
miR-423-5p expression	Low vs. High	0.715 (0.355-1.438)	0.346			
miR-200a-3p expression	Low vs. High	0.481 (0.234-0.985)	0.045	miR-200a-3p expression	0.364 (0.173-0.766)	0.008

Abbreviations: HR, hazard ratio; CI, confidence interval.

## DISCUSSION

In the present study, we identified the combination of let-7b-5p and miR-532-5p as the most suitable reference genes for urine miRNA detection by RT-qPCR.Normalized by the two reference genes, a seven-miRNA panel (miR-22-3p, miR-29a-3p, miR-375, miR-7-5p, miR-126-5p, miR-423-5p, miR-200a-3p) was designed as a novel diagnostic biomarker for BC based on a multivariate logistic regression model. Compared with traditional urine cytology, this panel was significantly superior based on its higher diagnostic accuracy. Furthermore, out of the seven miRNAs, miR-22-3p and miR-200a-3p were identified as independent factors for tumor recurrence in NMIBC. These findings suggest that urinary miRNAs obtained in a noninvasive manner may play important roles in the diagnosis and recurrence prediction of BC.

Current methods for BC diagnosis mainly depend on pathologic examination, which requires an invasive biopsy, cannot be repeated easily, and may miss early stage diseases. Therefore, the development of minimally invasive methods with high sensitivity and specificity, especially for the patients at early stages, is of urgent clinical need. Several publications have demonstrated that understanding the miRNA expression profile can help to improve the sensitivity and specificity of the diagnosis of cancer, such as hepatocellular cancer and esophageal squamous cell cancer [15–18]. MiRNAs have been proved to be abundant and stable in the circulation samples such as serum and urine [19, 20]. Our previous study has revealed a six-miRNA profile (miR-152, miR-148b-3p, miR-3187-3p, miR-15b-5p, miR-27a-3p and miR-30a-5p) in serum for BC detection via high-throughput MiSeq sequencing followed by two phases of RT-qPCR assays [21]. Unlike serum, urine may be a much specific and ideal source for finding biomarkers for BC, because it may pass through the malignant lesions in bladder before discharge and can be obtained in an absolutely noninvasive manner. However, little is known about the complete cell-free miRNA profiling in urine of BC. For this reason, in the present study, we described the global miRNA expression profile in urine and identified a diagnostic urinary miRNA signature for BC. Miseq sequencing is a high-throughput assay designed to initially screen global miRNA expression. Yet, considering individual variation, Miseq information from pooled urine samples could be potentially inaccurate. Thus, Miseq sequencing was performed on urine samples from 6 BC patients and 6 healthy donors. Candidate miRNAs revealed by sequencing were evaluated by two phases of RT-qPCR assays using a large number of individual samples. Finally, a seven-miRNA panel was designed for the diagnosis of BC with high accuracy. Comparison of the seven-miRNA panel with traditional urine cytology in the same cohort clearly demonstrated that the panel was a much more accurate indicator for BC, especially for early stage tumors (Ta and T1). Furthermore, because of the simplicity and reproducibility of obtaining urine sample, the investigation of the panel may be of great clinical interest as a routine applicable procedure.

At the urinary level, two out of seven miRNAs in our panel have been previously identified as differentially expressed in BC. Previous studies have demonstrated that the level of urinary miR-126 is significantly increased in BC than in controls [22, 23]. miR-200a has been reported to be down-regulated in urine of BC patients and has the potential to be developed as a noninvasive biomarker [12, 24]. These results reinforced our findings and further supported the use of urinary miRNAs as potential diagnostic indicators. Our study also showed the deregulation of miR-22-3p, miR-29a-3p, miR-375, miR-423-5p and miR-7-5p in urine of BC for the first time. Nevertheless, little was known about the expression levels of urinary miRNAs in recurrent NMIBC cases and their predictive potential. Because all NMIBC patients in the present study were initially diagnosed, further studies are needed to validate whether the diagnostic miRNA panel could perform efficiently in recurrent NMIBC. In addition, several miRNAs in our panel were involved in general tumorigenesis at the tissue level. For instance, the miR-200 family is strongly associated with a pathologic epithelial to mesenchymal transition (EMT) and impose strong effects on cancer cell proliferation, migration, invasion and metastasis [25–27]. Wiklund *et al*. have demonstrated that the miR-200 members are silenced in MIBC and has been implicated in tumor invasion by targeting the transcriptional repressors of E-cadherin, ZEB1 and ZEB2 [28]. The ectopic expression of miR-29a promotes angiogenesis and tumor cell proliferation through the down-regulation of anti-angiogenic genes such as Col4a2, Spry1 and Timp3 [29]. Moreover, Jia and colleagues have shown that miR-126 inhibits invasion in BC through the downregulation of ADAM9 expression [30]. In addition, low miR-7 expression in Ta tumor has been shown to be in line with the frequent activation of FGFR3 signaling, and miR-7 is an important member of the miRNA signature for FGFR3 mutated cases in BC [31]. Although the complex mechanism that regulates the biogenesis of these miRNAs in BC still remain unknown, these functional studies of miRNAs in tumor tissues may be helpful for evaluating cell-free miRNAs in urine as effective noninvasive biomarkers for BC detection.

Previous studies have shown that the deregulation of miRNA expression in BC tissues significantly correlates with tumor recurrence and progression [32, 33]. The clinical significance of our findings could be extended if the expression levels of diagnostic miRNAs convey prognostic information. With this in mind, we further investigated whether miRNAs revealed in this study could be used as potential indictors for recurrence of BC. Interestingly, after resection of tumors, the levels of previously increased miR-22-3p and miR-29a-3p in urine were significantly down-regulated, implicating a strong association between their levels and the tumor status of the patients. Taking a step further, Cox proportional hazards regression model analysis revealed that miR-22-3p and miR-200a-3p were independent factors influencing recurrence of NMIBC. In agreement with our findings, miR-200a was identified as an independent predictor of NMIBC recurrence by Yun *et al* [12]. Thus, we speculated that pretreatment urinary levels of miR-22-3p and miR-200a-3p might help to identify NMIBC at high or low risks for tumor recurrence.

Compared with other studies of miRNA in BC diagnosis, our study was unique owing to reasons as follows: specific selection of aberrantly expressed miRNAs by genome-wide urine miRNA profiles via MiSeq sequencing rather than from lists of deregulated miRNAs from previous literatures, combination of seven miRNAs in a multivariate logistic regression model instead of single miRNA, confirmation with independent validation, large number of urine samples analyzed, direct comparison with urine cytology in the same cohort. In addition, the identification of suitable reference gene could account for this increased performance. Currently, there is no standard endogenous control for urinary miRNA studies. In the present study, a three-step approach with MiSeq sequencing, reference miRNA selection through computer software, and RT-qPCR validation was used. Results of geNorm and NormFinder analyses revealed that a combination of let-7b-5p and miR-532-5p could be used as the most stable pair of reference genes. However, our study also has limitations. As our study only included BC patients, specificity of the miRMA panel for patients with BC was still unknown. Among the seven miRNA identified in the present study, some have been reported to be associated with kidney injury in studies using urine samples [34–38]. As patients with renal insufficiency were excluded in this study, it is not clear whether the panel could discriminate patients with diseases affecting kidneys or urinary tract. This current study was inadequate to bring the assay to the clinic. Therefore, our findings are needed to be validated in larger cohorts from different centers before be clinically use. Further studies focusing on the specificity of our findings in clinic are needed.

In conclusion, we have defined the distinctive urinary miRNA signatures for BC diagnosis and recurrence prediction. Further multi-center studies, including more patients enrollment from several hospitals or even more diverse ethnic populations, are required to confirm whether the results of the present study can be incorporated into clinical routine.

## MATERIALs AND METHODS

### Study design, patients and control subjects

A total of 276 BC patients and 276 control individuals were recruited from Qilu Hospital, Shandong University between January 2005 and May 2009. The present study was divided into two parts. All participants in different phases were randomly allocated. In the first part, reference genes for RT-qPCR assays were identified by systematic analysis. RNAs in the urine samples from 6 BC patients and 6 healthy donors were sequenced on a MiSeq sequencing platform (Illumina). The candidate reference miRNAs were further assessed in 143 BC patients and 143 controls. In the second part, validation of potential diagnostic miRNAs were conducted in the following two phases. In the training phase, the expression of the selected miRNAs were measured by RT-qPCR assays in a cohort of 150 BC patients and 150 controls, and a diagnostic miRNA panel was constructed based on a logistic regression model for the differentiation between the BC group and the control group. In the validation phase, the parameters of the logistic model identified from the training phase were applied to another independent cohort of 120 BC patients and 120 controls for validating the diagnostic performance of the constructed algorithm. Meanwhile, urine cytology was conducted on the same cohort. Additionally, postoperative urine samples (10 days after transurethral resection or radical cystectomy) were collected from 21 BC patients, from whom matching preoperative urine samples were available for determining whether miRNA expression was altered subsequent to tumor resection. Patients with BC were followed up at regular intervals until recurrence or June 30, 2014. The median follow-up time was 61 months (range: 5-76 months). Fourteen patients with BC were excluded because of incomplete follow-up information.

Diagnosis of BC was confirmed by histopathology or histobiopsy. All NMIBC patients were initially diagnosed with nonmuscle-invasive diseases. All MIBC patients were initially diagnosed with muscle-invasive diseases. Tumor stage was defined according to the 2002 UICC TNM classification of BC and tumor grade was designated according to the WHO 2004 grading scheme. Control participants without history of BC were recruited from a large pool of individuals seeking a routine health checkup at the Healthy Physical Examination Centre of Qilu Hospital, Shandong University. People who showed no evidence of disease were selected as tumor-free controls. Participants with diabetes mellitus or renal insufficiency were excluded from this study. There was no significant difference in the distribution of age, sex and tumor characteristics between BC and control groups (Supplementary Table 5). The investigational protocol was approved by the Clinical Research Ethics Committee of Qilu Hospital, Shandong University and written informed consent was obtained from each participant.

### Urine preparation

Urine samples were collected in the morning prior to any therapies at the day before radical cystectomy and/or TUR. For miRNA analysis, 5 mL midstream urine was immediately taken to our laboratory and stored at 4°C. Then urine was centrifuged at 1,500 × g for 10 min at 4°C within 2 h of collection, followed by a second centrifugation at 13,800 × g for 15 min at 4°C to eliminate residual cell debris. The supernatant urine was then stored at -80°C till use. Meanwhile, 15 ml midstream urine was centrifuged at 1,300 × g for 10 min and sediments were then processed for cytological examination in a blinded fashion by two cytopathologists.

### MiSeq sequencing

Equal volumes of urine from 6 BC patients and 6 healthy donors were subjected to MiSeq sequencing. Total RNA of each sample was used to prepare the miRNA sequencing library using NEBNext®Multiplex Small RNA Library Prep Set for Illumina® (New England Biolabs). After quantified on an Agilent 2100 Bioanalyzer, the library was denatured with 0.1M NaOH to generate single-stranded DNA molecules, which were captured on flow cells, amplified *in situ* and sequenced on MiSeq according to the manufacturer's instruction (Illumina).

Image analysis and base calling were performed by an off-line basecaller software (OLB V1.8.0). Subsequently, index sequences were trimmed from clean reads (reads that passed Solexa CHASTITY quality filter) and the reads shorter than 8 nt were discarded. Then, reads passing filter (length≧15 nt) were mapped to the latest human reference miRNA precursor set (Sanger miRBase 17.0) using the Novoalign software(v2.07.11). Differentially expressed miRNA was determined using a fold change filtering (larger than 2-fold change, p < 0.05).

### RT-qPCR analysis of urinary miRNAs

Total RNA was isolated from urine supernatant (200 μl) using miRCURY™ RNA Isolation Kit-Biofluids (Exiqon, Vedbaek, Denmark) according to the manufacturer's instructions. Total RNA concentration was measured on a NanoDrop spectrophotometer (Thermo Fisher Scientific, Waltham, MA). First-strand cDNA was generated from about 100 ng of RNA using the Takara SYBR^®^ PrimeScript™ miRNA RT-PCR Kit (Takara Bio Inc) in a final volume of 20 μl reverse transcription (RT) reaction system by following the protocol provided by the manufacturer. The RT condition was set as follows: 37°C for 60 min, 85°C for 5 sec and 4°C for 60 min. cDNA synthesis was performed in triplicate to ensure enough quantity for qPCR assays. Then, 2 μl of 5-fold diluted cDNA was added in a qPCR reaction consisting of 12.5 μl SYBR Premix Ex Taq II, 0.5 μl Dye II 2 μl of 5 μM forward primer, 1 μl of 10 μM Uni-miR RT-qPCR Primer, and 7 μl of ddH_2_O. The cycling conditions were 95°C for 30 sec, 45 cycles of 95°C for 5 sec and 57°C for 34 sec. All reactions in triplicate were assessed in the ABI PRISM 7500 Sequence Detection System (Applied Biosystems, Foster City, CA). The relative expression fold change was calculated by using the 2^-ΔΔCt^ method [39].

### Identification of endogenous references

To determine suitable miRNA references for RT-qPCR, we firstly selected candidate reference miRNAs via MiSeq sequencing. The usefulness of potential miRNAs together with U6 was tested in a cohort of 80 BC patients and 80 controls by geNorm and NormFinder software [40–43].To further validate the stability ofidentified reference genes, another cohort of 63 BC patients and 63 controls were recruited.

### Statistical analysis

The selected reference genes for normalization were evaluated by the geNorm and NormFinder software. Mann-Whitney U test was performed to test the differences of miRNAs expression between unpaired groups. The Wilcoxon test was used to compare miRNA expression in paired urine samples obtained before surgical tumor resection and 4 weeks after tumor resection. Receiver operating characteristic (ROC) curves were established to discriminate BCs from controls by using MedCalc 9.3.9.0 (MedCalc, Mariakerke, Belgium). Logistic regression analysis was performed using the Matlab software (Matlab, R2013a). Kaplan–Meier analysis with log-rank test was used for survival curves. The Cox proportional hazard regression model was performed to determine the independent prognostic factors. Other analyses were conducted in SPSS version 17.0 software (SPSS Inc., Chicago, IL) and difference with *p* < 0.05 was considered as significant.

## SUPPLEMENTARY MATERIALS FIGURES AND TABLES


