# Supplementary material for: Cell-free microRNA expression signatures in urine serve as novel noninvasive biomarkers for diagnosis and recurrence prediction of bladder cancer
Source: Oncotarget. 2017 Mar 28;8(25):40832–42. doi: 10.18632/oncotarget.16586 (PMC5522322; doi:10.18632/oncotarget.16586)
Supplement: Supplementary file 1 [file oncotarget-08-40832-s001.pdf]

Cell-free microRNA expression signatures in urine serve as novel noninvasive biomarkers for diagnosis and recurrence prediction of bladder cancer

SUPPLEMENTARY MATERIALS

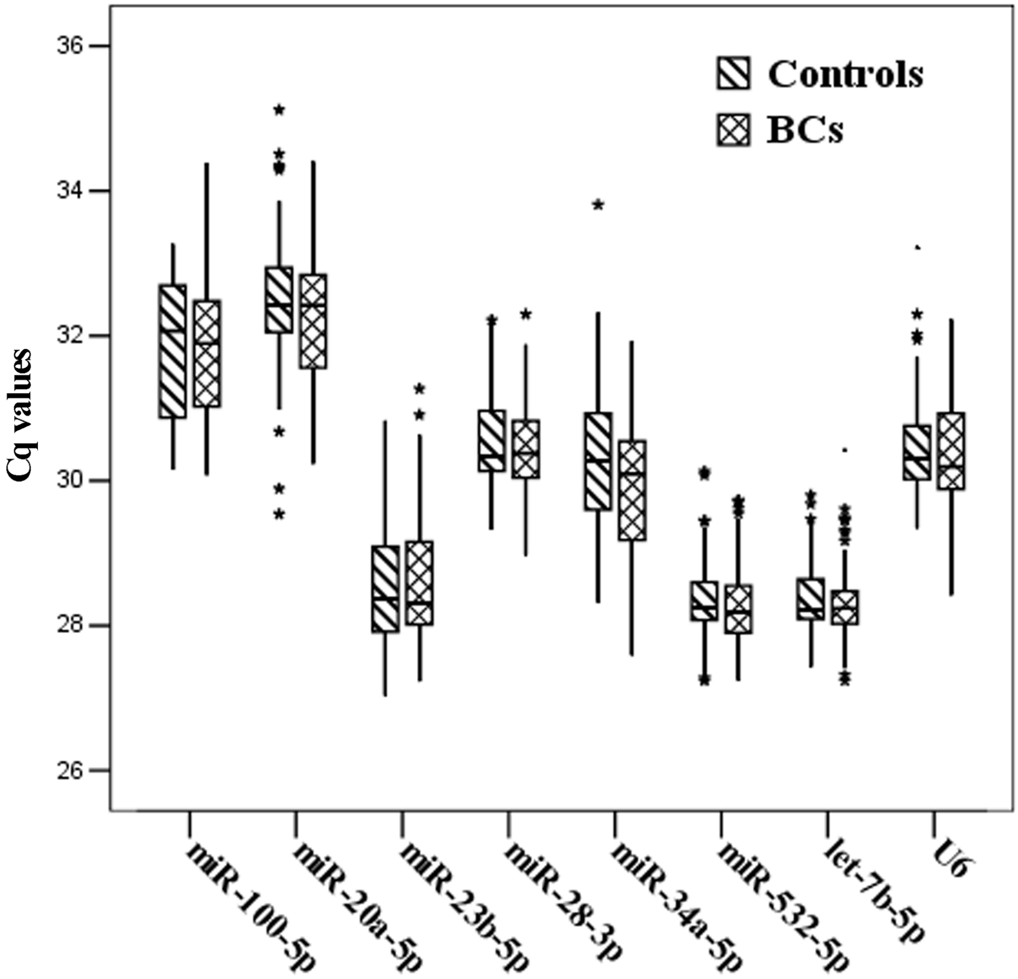

Supplementary Figure 1: Cq values of candidate reference genes. Cq values of candidate reference genes in BCs and controls. No significant difference was found between the two groups (p>0.05). The bottom and the top of the box represent P_{25} an d P_{75}. The line inside the box represents P_{50}. The whiskers below and above the box indicate the quantitative values between P_{25} and the minimum value not including abnormal values, and the quantitative values between P_{75} and maximum value except abnormal values, respectively.

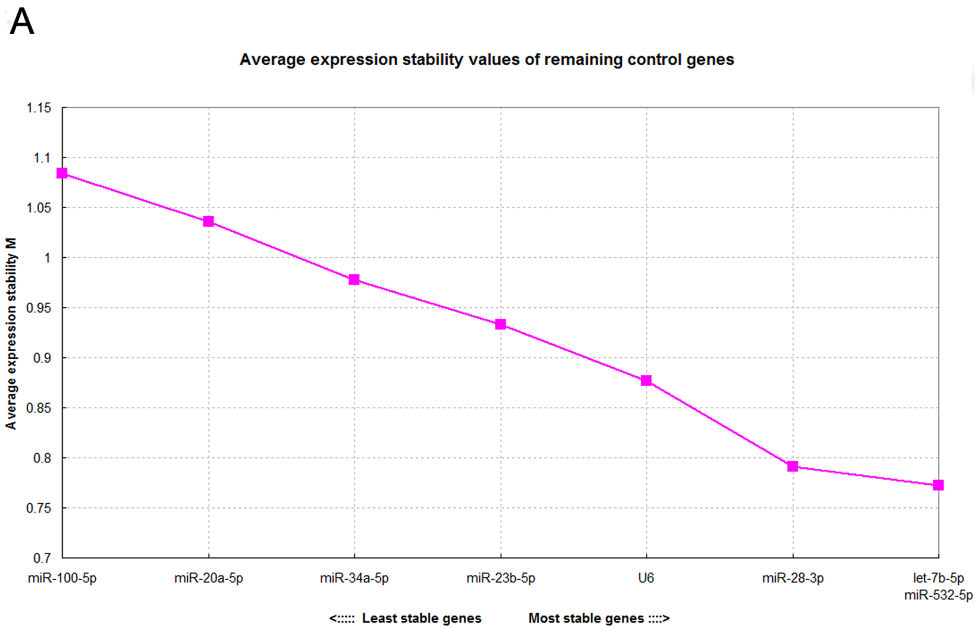

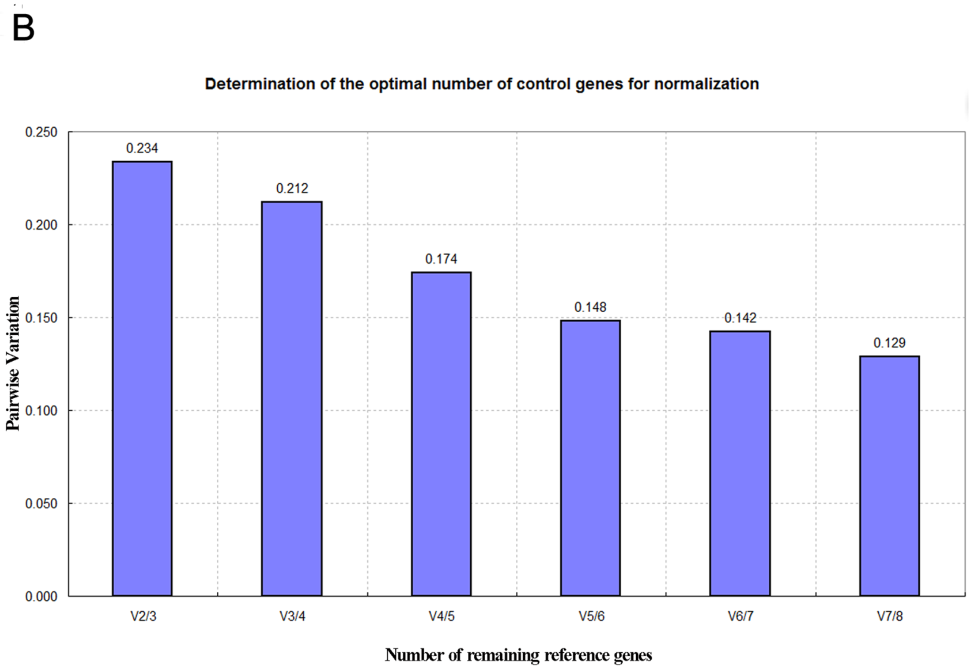

Supplementary Figure 2: GeNorm analysis of candidate reference genes. **(A)** Ranking of candidate reference genes according to average stability. Genes with the lowest M value had the most stable expression. The x-axis represents the ranking of candidate reference genes in order of increasing stability from left to right. **(B)** Determination of optimal number of reference genes for normalization. The geNorm software calculated a normalization factor assessing the optimal number of reference genes for generating that factor. The cut-off value was VNF<0.15 and the optimal number of reference genes was five (V5/6). GeNorm analysis showed that optimal normalization of gene expression could be achieved using the top five most stable reference genes (let-7b-5p, miR-532-5p, miR-28-3p, U6 and miR-23b-5p) in relation to six genes as shown in **(A)**.

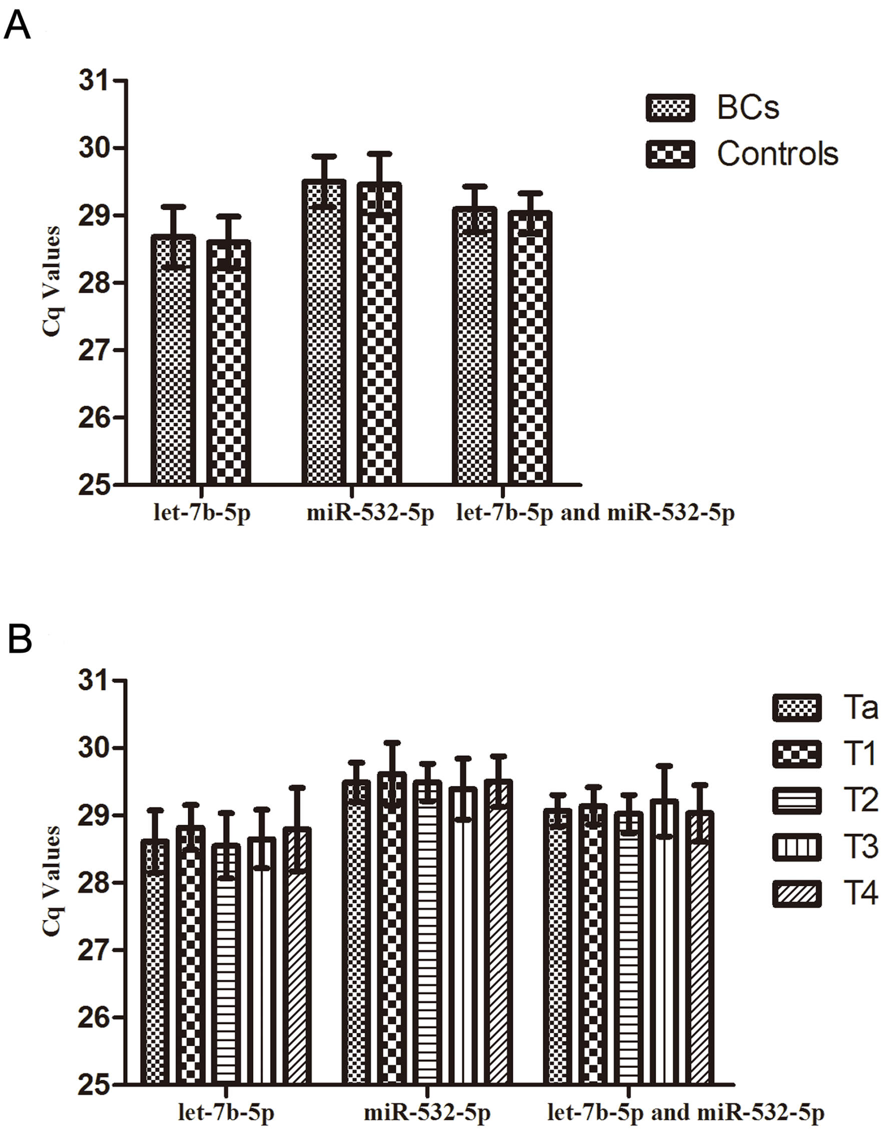

Supplementary Figure 3: The Cq values of let-7b-5p, miR-532-5p, and the combination of let-7b-5p and miR-532-5p in two groups and different stages of BC. No significant difference was found among the 3 reference genes in the two groups **(A)** and among different stages of BC **(B)** (both $p>0.05$).

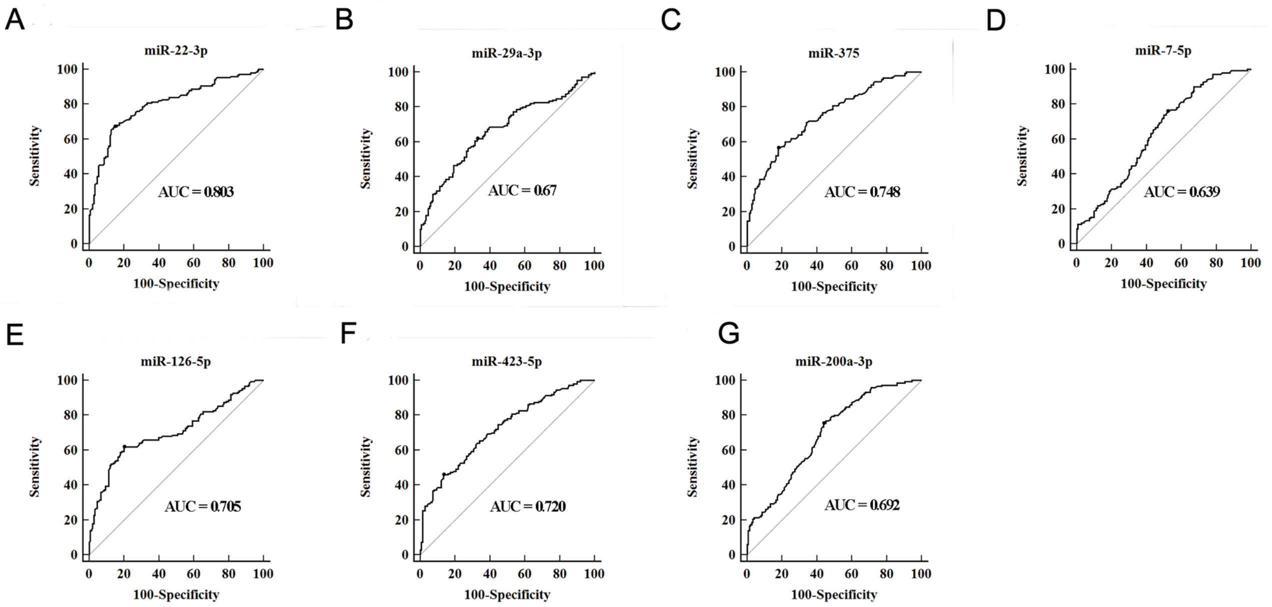

Supplementary Figure 4: Diagnostic performance of selected lncRNAs for BC patients versus controls. ROC curves analysis for the detection of BC using miR-22-3p **(A)**, miR-29a-3p **(B)**, miR-375 **(C)**, miR-7-5p **(D)**, miR-126-5p **(E)**, miR-423-5p **(F)**, miR-200a-3p **(G)** in patients with BC (n = 150) and control individuals (n = 150) in training set.

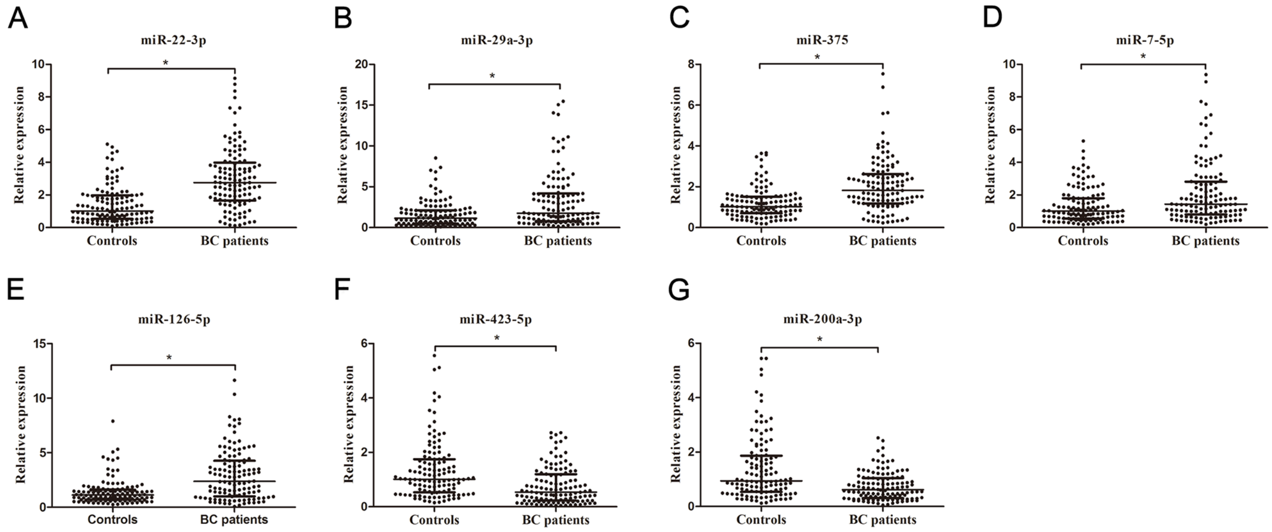

Supplementary Figure 5: Relative expression of seven selected miRNAs in urine. The relative expressions of seven selected urinary miRNAs in patients with BC (n = 120) and control individuals (n = 120) using RT-qPCR assay in validation set **(A-G)**, *p< 0.001.

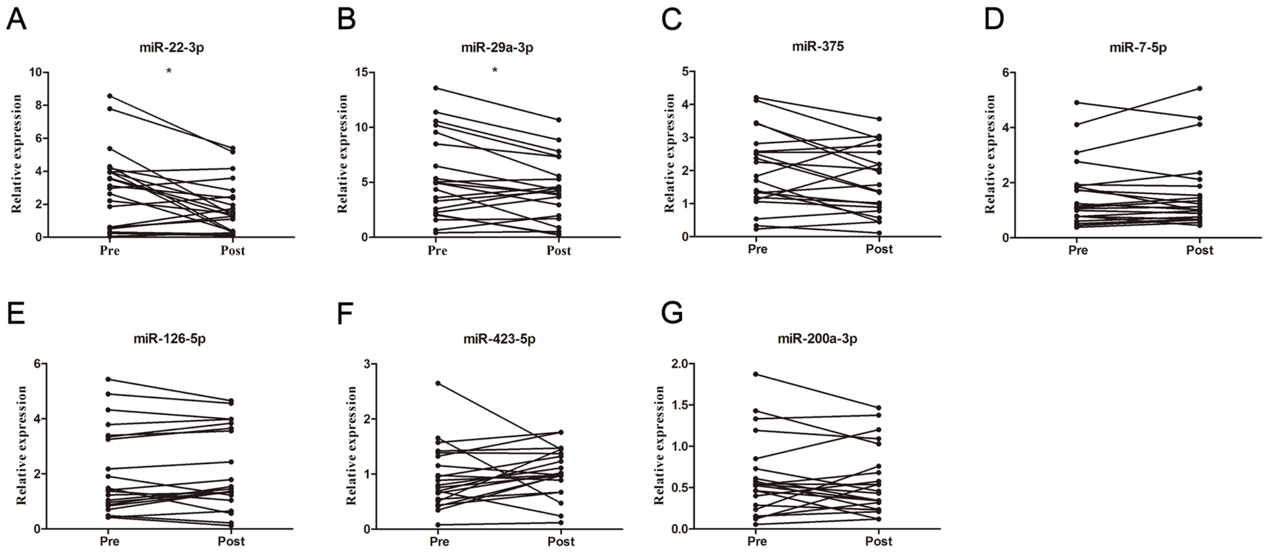

Supplementary Figure 6: Expression levels of selected miRNAs in BC patients before surgery and ten days after surgery. Expressions of miR-22-3p **(A)**, miR-29a-3p **(B)**, miR-375 **(C)**, miR-7-5p **(D)**, miR-126-5p **(E)**, miR-423-5p **(F)**, miR-200a-3p **(G)** in urine of patients with BC after tumor resection.

Supplementary Table 1: Candidate reference genes determined by Miseq sequencing

Candidate reference gene	BC	Control	BC/Control
let-7b-5p	12762	11748	1.09
miR-17-5p	367	431	0.85
miR-20a-5p	513	434	1.18
miR-23b-5p	183	154	1.19
miR-28-3p	2200	1871	1.18
miR-30b-5p	512	551	0.93
miR-34a-5p	61	52	1.17
miR-98-5p	843	810	1.04
miR-99b-5p	293	323	0.91
miR-100-5p	2054	1724	1.19
miR-133a-3p	367	352	1.04
miR-194-5p	794	890	0.89
miR-532-5p	277	312	0.89

Supplementary Table 2: Altered miRNAs in BC and control urine samples determined by Miseq sequencing

Up-regulated miRNA	BC	Control	BC/Control	Down-regulated miRNA	BC	Control	BC/Control
let-7e-5p	843	291	2.90	miR-10a-3p	61	183	0.33
let-7i-5p	2164	758	2.85	miR-106b-5p	110	521	0.21
miR-7-5p	587	229	2.56	miR-200a-3p	686	6124	0.11
miR-22-3p	114342	15464	7.39	miR-423-5p	1156	5427	0.21
miR-23a-3p	293	62	4.73	miR-429	73	383	0.19
miR-29a-3p	587	114	5.15	miR-455-5p	61	152	0.40
miR-125b-5p	1577	332	4.75	miR-505-3p	103	287	0.36
miR-126-5p	147	51	2.88				
miR-148a-3p	6454	1527	4.23				
miR-184-5p	631	121	5.21				
miR-181b-5p	293	140	2.09				
miR-186-5p	1614	166	9.72				
miR-221-3p	1504	741	2.03				
miR-375	33151	3012	11.0				
miR-574-5p	550	81	6.79				
miR-941	147	52	2.83				

Supplementary Table 3: Ranking and best combination of candidate reference genes based on expression stability calculated by NormFinder and geNorm

Rank	NormFinder		geNorm	
	gene	stability	gene	stability (M)
1	let-7b-5p	0.050	let-7b-5p	0.917
2	miR-532-5p	0.057	miR-532-5p	0.953
3	U6	0.074	miR-28-3p	1.032
4	miR-23b-5p	0.079	U6	1.080
5	miR-34a-5p	0.083	miR-34a-5p	1.117
6	miR-28-3p	0.087	miR-23b-3p	1.128
7	miR-100-5p	0.105	miR-20a-5p	1.214
8	miR-20a-5p	0.125	miR-100-5p	1.229
Best combination	let-7b-5p/miR-532-5p	0.038	let-7b-5p/miR-532-5p	0.773

Supplementary Table 4: Univariate Cox proportional hazards regression model analysis of recurrence-free survival in MIBC patients in validation set

Parameters	Categories	Univariate analysis	
		HR (95% CI)	*p* Value
miR-22-3p expression	Low vs. High	0.630 (0.200-1.987)	0.431
miR-29a-3p expression	Low vs. High	0976 (0.315-3.025)	0.966
miR-375 expression	Low vs. High	2.139 (0.644-7.107)	0.215
miR-7-5p expression	Low vs. High	0.898 (0.289-2.784)	0.852
miR-126-5p expression	Low vs. High	1.413 (0.448-4.455)	0.555
miR-423-5p expression	Low vs. High	0.588 (0.186-1.853)	0.364
miR-200a-3p expression	Low vs. High	1.273 (0.404-4.013)	0.680

Abbreviations: HR, hazard ratio; CI, confidence interval.

Supplementary Table 5: Characteristics of study participants in training set and validation set

Variable	Training set (n=300)	Validation set (n=240)	*p*
**Control (number)**	150	120	
**Age (years)**	66 (59-73)	66 (58-73)	0.60
**Sex**			0.78
Male	109 (72.67%)	89 (74.17%)	
Female	41 (27.33%)	31 (25.83%)	
**BC (number)**	150	120	
**Age (years)**	68 (60-75)	67 (60-74)	0.52
**Sex**			0.73
Male	111 (74.00%)	91 (75.83%)	
Female	39 (26.00%)	29 (24.17%)	
**Tumor stage**			0.97
Ta	34 (22.67%)	25 (20.83%)	
T1	51 (34.00%)	43 (35.83%)	
T2	22 (14.67%)	19 (15.83%)	
T3	25 (16.67%)	21 (17.50%)	
T4	18 (12.00%)	12 (10.00%)	
**Tumor grade**			0.91
Low grade	86 (57.33%)	68 (56.67%)	
High grade	64 (42.67%)	52 (43.33%)	
**Lymph node metastasis**			0.84
Negative	130 (86.67%)	103 (85.83%)	
Positive	20 (13.33%)	17 (14.17%)	
